# Psychometric Evaluation of the Serbian Version of the Southampton Dupuytren’s Scoring Scheme in Patients with Dupuytren’s Contracture

**DOI:** 10.3390/jcm14217528

**Published:** 2025-10-24

**Authors:** Milos Vucetic, Vedrana Pavlovic, Ksenija Markovic, Suzana Milutinovic, Nikolina Stanimirovic, Luka Joksimovic, Aleksandar Matejic, Bojan Petrovic, Nemanja Jovanovic, Nikola Bogosavljevic, Dejan Aleksandric, Draško Vasovic, Filip Pilipovic, Danijela Radulovic, Milan Stojcic, Natasa Milic

**Affiliations:** 1 Institute for Orthopedic Surgery “Banjica”, 11000 Belgrade, Serbia; 2 Institute for Medical Statistics and Informatics, Faculty of Medicine, University of Belgrade, 11000 Belgrade, Serbia; 3 Clinic for Orthopedic Surgery and Traumatology, University Clinical Center of Serbia, 11000 Belgrade, Serbia; 4 Faculty of Medicine, University of Belgrade, 11000 Belgrade, Serbia; 5 Clinic for Burns, Plastic and Reconstructive Surgery, University Clinical Center of Serbia, 11000 Belgrade, Serbia; 6 Department for Primary Health Care and Public Health, Faculty of Medicine Foca, University of East Sarajevo, 71123 East Sarajevo, Bosnia and Herzegovina

**Keywords:** Dupuytren’s Contracture, Southampton Dupuytren’s scoring scheme, patient-reported outcome measures, psychometric validation, reliability, validity, cultural adaptation

## Abstract

**Background/Objectives**: Dupuytren’s contracture is a chronic fibroproliferative disorder of the palmar fascia that leads to progressive flexion deformities and functional impairment. The Southampton Dupuytren’s Scoring Scheme (SDSS) is a disease-specific patient-reported outcome measure designed to quantify disability in this condition. This study aimed to translate, culturally adapt, and evaluate the psychometric properties of the Serbian version of the SDSS. **Methods**: A cross-sectional study was conducted at the Institute for Orthopedic Surgery “Banjica”, Belgrade, from January 2024 to March 2025. Sixty-eight patients with Dupuytren’s contracture completed the Serbian SDSS, the Disabilities of the Arm, Shoulder and Hand (DASH) questionnaire, the 12-Item Short Form Health Survey (SF-12), and a Visual Analogue Scale (VAS) for pain. Translation followed standardized forward–backward procedures. Internal consistency was assessed with Cronbach’s alpha, construct validity with confirmatory factor analysis (CFA), and convergent validity with Pearson’s correlation coefficients. **Results**: The Serbian SDSS demonstrated excellent internal consistency (Cronbach’s α = 0.914). CFA supported a unidimensional five-item structure with strong factor loadings (0.76–0.93) and acceptable fit indices (χ^2^ = 10.094, df = 5, *p* = 0.073; IFI = 0.979; CFI = 0.978; TLI = 0.956). Convergent validity was confirmed by strong correlations with DASH (r = 0.779) and VAS (r = 0.702) and a strong negative correlation with SF-12 PCS (r = −0.802). **Conclusions**: The Serbian SDSS is a valid and reliable instrument for assessing functional disability in patients with Dupuytren’s contracture and offers a robust, patient-centered measure for clinical and research use.

## 1. Introduction

Dupuytren’s contracture is a chronic, progressive fibroproliferative disorder of the palmar fascia, characterized by aberrant activation of myofibroblasts and excessive deposition of collagen [1]. These pathological changes lead to the formation of nodules and fibrous cords that progressively shorten the fascia, resulting in fixed flexion deformities of the fingers and loss of extension [2,3]. The condition predominantly affects individuals of Northern European descent, with the highest prevalence observed in men over the age of 50, and population-based studies report an overall prevalence ranging from approximately 2% to 5% in the United Kingdom, increasing with age and male gender [4,5,6]. Recent epidemiological data further indicate that the annual incidence lies between about 1.4 and 1.7 cases per 1000 person-years, while the overall prevalence is close to 2%, with both measures rising with age and reaching their highest levels among men between 61 and 80 years of age [7]. Established risk factors include genetic predisposition, lifestyle factors such as alcohol consumption and smoking, as well as comorbidities, including diabetes mellitus and epilepsy [6]. Clinical manifestations range from painless nodules to debilitating flexion contractures of the fingers, which can significantly impair hand function [8,9]. Management strategies for Dupuytren’s contracture are determined by disease stage and functional impact. Non-surgical interventions such as collagenase Clostridium histolyticum (CCH) injections, low-dose radiotherapy, and intralesional corticosteroid administration represent effective alternatives to surgery, particularly in early disease stages [10,11]. Surgical approaches, including percutaneous needle fasciotomy and limited fasciectomy, remain the gold standard for advanced cases, offering durable correction but carrying the drawbacks of prolonged recovery and recurrence risk [12]. Emerging therapeutic modalities, including anti-tumor necrosis factor (anti-TNF) agents and interleukin-33 (IL-33) inhibitors aim to target the underlying molecular mechanisms of fibrosis and hold promise for advancing disease management in the future [13,14]. The assessment of treatment outcomes in Dupuytren’s contracture has traditionally relied on physician-based measures such as joint angles, recurrence rates and grip strength [15]. While these objective parameters provide valuable clinical information, they fail to capture the broader impact of the disease on patients’ functional abilities and quality of life. The development of patient-reported outcome measures (PROMs) specifically adapted to Dupuytren’s contracture represents a major advancement, enabling evaluation of treatment effectiveness from the patient’s perspective and offering insights into satisfaction, disability and daily functional challenges [16,17]. Although widely used instruments such as the URAM scale and the Quick Disabilities of the Arm, Shoulder and Hand (QuickDASH) questionnaire remain valuable, they have notable limitations in capturing the psychosocial and cultural dimensions of the disease [18,19,20]. To address these shortcomings, the Southampton Dupuytren’s Scoring Scheme (SDSS) was developed as a condition-specific outcome tool. Introduced and validated in 2014, the SDSS condenses the functional impact of the disease into five domains, each scored from 0 (no problem) to 4 (severe problem), producing a total score ranging from 0 to 20, with higher scores reflecting greater disability [16]. Validation studies confirmed the strong psychometric properties of the instrument, demonstrating good internal consistency (Cronbach’s alpha = 0.87) and high test–retest reliability (r = 0.79). Since its initial development, the SDSS has been translated and validated in several European populations, including Danish and other Northern European cohorts, and has been widely adopted as a standard patient-reported outcome measure for monitoring functional disability in Dupuytren’s disease [7,21]. These international validations further support its robustness and highlight the need for culturally adapted versions such as the present Serbian translation. The clinical and research implications of the SDSS are considerable. In practice, it enables clinicians to quantify functional disability, monitor disease progression, and tailor treatment planning to the individual needs of patients. Since the SDSS questionnaire had not been validated in the Serbian population, the aim of this study was to assess the psychometric properties of the Serbian version of the SDSS by determining its construct and convergent validity, as well as its internal consistency.

## 2. Materials and Methods

A cross-sectional study was carried out at the Institute for Orthopedic Surgery “Banjica”, Belgrade, Serbia, between January 2024 and March 2025, included patients diagnosed with Dupuytren’s contracture. Participants completed the questionnaires during routine clinical visits at the Institute for Orthopedic Surgery “Banjica”. Data were collected in paper-and-pencil format by trained research staff and subsequently entered into a secure database for analysis. Ethical approval was granted by the Ethics Committee of the Institute for Orthopedic Surgery “Banjica” (reference number: i-113/7; date: 3 April 2023). Participation was voluntary, signed written informed consent was obtained, and patients’ data were treated with strict confidentiality.

Study Instruments

The research instruments included self-reported questionnaires covering socio-demographic data (age and gender), the Southampton Dupuytren’s Scoring Scheme (SDSS), the Disabilities of the Arm, Shoulder and Hand (DASH) questionnaire, the 12-Item Short Form Health Survey (SF-12), and the Visual Analogue Scale (VAS) for pain.

SDSS is a disease-specific, patient-reported outcome measure developed in 2014 to assess disability caused by Dupuytren’s disease. The instrument was developed in stages, following the recommendations of the Derby Outcomes Conference, and was validated against established instruments [16,22]. The questionnaire comprises five items addressing discomfort, personal activities (e.g., washing face, dressing, washing hands, washing hair, putting on gloves), domestic activities (e.g., holding a glass or cup, opening jars, eating, cooking), work/social interaction (e.g., using the computer, writing, shaking hands, cosmetic appearance), and hobbies (e.g., driving or cycling, racket sports, DIY, playing musical instruments, gardening). Items are scored on a five-point Likert scale, where 0 indicates no problem and 4 represents severe problem. The total score ranges from 0 to 20, with higher values reflecting greater disability. The SDSS is self-administered and requires approximately one minute to complete, making it a practical tool for clinical practice and research in Dupuytren’s disease [16].

The DASH questionnaire is a self-report instrument created to evaluate physical function and symptoms in individuals with musculoskeletal disorders of the upper limb. It includes 30 items, each scored on a five-point Likert scale. A score of 1 denotes no difficulty or symptoms, whereas a score of 5 indicates severe symptoms or inability to perform activity. The total score is derived by summing the item responses and converting the result to a scale from 0 to 100, where 0 represents no disability and 100 represents the most severe disability. If more than three items are left unanswered, the DASH total score is considered invalid and cannot be computed [23].

SF-12 is a widely used, generic, self-administered patient-reported outcome measure for assessing health-related quality of life. The questionnaire includes 12 items. These items capture information across eight domains: physical functioning (PF), role physical (RP), bodily pain (BP), general health (GH), vitality (VT), social functioning (SF), role emotional (RE), and mental health (MH). From these domains, two summary scores are calculated using the norm-based scoring algorithm recommended by Ware: the Physical Component Summary (PCS-12) and the Mental Component Summary (MCS-12). Item responses are expressed on ordinal scales (e.g., excellent to poor, always to never) or dichotomous scales (yes/no). Both PCS and MCS are standardized with a mean value of 50 and a standard deviation of 10, where higher scores indicate a better health-related quality of life [24].

VAS is a simple, self-administered, widely used instrument for assessing the intensity of pain. It consists of a 10-cm horizontal line, with descriptors at each end representing the extremes of pain intensity: 0 corresponds to no pain, while 10 denotes the worst pain imaginable. Participants are instructed to place a mark on the line corresponding to the intensity of pain they experienced. The score is determined by measuring the distance in centimeters from the “no pain” anchor to the participant’s mark, yielding a continuous variable ranging from 0 to 10 [25].

Translation and Cultural Adaptation

Permission to translate and use the SDSS was obtained from the original author. The Serbian version was developed using the standard forward–backward translation procedure in line with international guidelines [26]. Two independent native Serbian speakers performed forward translations, which were reconciled into a single version. A native English speaker, blinded to the original instrument, performed the back-translation. Discrepancies between the original and back-translated versions were reviewed by an expert panel consisting of clinicians and methodologists, and resolved by consensus. Before finalization, the preliminary version of the questionnaire was piloted on a small group of patients with Dupuytren’s contracture. This step was undertaken to ensure the clarity and comprehensibility of the items. Minor wording adjustments were made based on feedback, resulting in the final Serbian version of the SDSS used in this study.

Statistical analysis

Descriptive statistics were used to characterize the study sample. Numerical variables were reported as means or medians with corresponding measures of variability. Categorical variables were presented as absolute numbers and percentages. The psychometric properties of the SDSS were evaluated through the examination of construct and convergent validity and internal consistency (reliability). Internal consistency for the entire scale and its subscales was analyzed using Cronbach’s alpha coefficient, with values ≥ 0.70 considered acceptable [27]. Confirmatory factor analysis (CFA) was carried out with maximum likelihood estimation to test the original single factor structure of the SDSS. Model fit was assessed using the chi-square test, with *p*-values > 0.05 suggesting an adequate fit. Additional indices included the comparative fit index (CFI), the incremental fit index (IFI), Tucker–Lewis index (TLI) and the root mean square error of approximation (RMSEA). Thresholds for acceptable fit were set at CFI, IFI, and TLI > 0.90, and RMSEA < 0.06. Pearson’ s correlation coefficients were used to explore associations between SDSS scores and the DASH, VAS and the two SF-12 subscales. According to Evans’ classification, correlations were interpreted as very weak (<0.20), weak (0.20–0.39), moderate (0.40–0.59), strong (0.60–0.79), and very strong (≥0.80) [28]. All analyses were two-tailed and statistical significance was set at *p* < 0.05. Statistical analysis was performed using Amos 21 (IBM SPSS Inc., Chicago, IL, USA) and IBM SPSS Statistics version 29 (IBM SPSS Inc., Chicago, IL, USA).

## 3. Results

The study included 68 participants who completed the SDSS questionnaire. Their mean age was 64.6 ± 9.5 years and the majority were male (80.9%). The mean SDSS total score was 7.1 (95% CI: 5.7–8.5), and the mean DASH score was 26.5 (95% CI: 20.2–32.9). For health-related quality of life, the mean SF-12 PCS score was 47.0 (95% CI: 44.4–49.7) and the mean SF-12 MCS score was 42.1 (95% CI: 40.3–43.9). Regarding VAS pain, the median score was 2.0 (IQR: 0.9–4.3) (Table 1).

Participants’ responses to the SDSS items are presented in Table 2. Trouble with discomfort was most often reported as “minor inconvenience” (36.8%), while 7.4% reported a severe problem. For trouble with personal activities (e.g., washing face, dressing, washing hands, washing hair, putting on gloves), the most common response was “modest inconvenience” (23.5%), whereas 14.7% of participants reported a severe problem. When asked about trouble while performing domestic activities (e.g., holding a glass or cup, opening jars, eating, cooking), “minor inconvenience” was most frequent (26.5%), and 8.8% of study participants reported a severe problem. For trouble during work/social interaction (e.g., using the computer, writing, shaking hands, cosmetic appearance), 11.8% participants reported a severe problem, while majority of respondents (38.2%) did not experience a problem during these interactions. Regarding trouble with hobbies (e.g., driving or cycling, racket sports, DIY, playing musical instruments, gardening), the majority of participants reported “minor inconvenience” (38.2%), followed by “no problem” (29.4%), with 14.7% reporting a severe problem (Table 2).

Table 3 presents the results of the internal consistency analysis for the Serbian version of SDSS questionnaire. The SDSS questionnaire, analyzed as a single-factor structure with five items, demonstrated excellent reliability (Cronbach’s alpha = 0.914) (Table 3).

**Figure 1:**
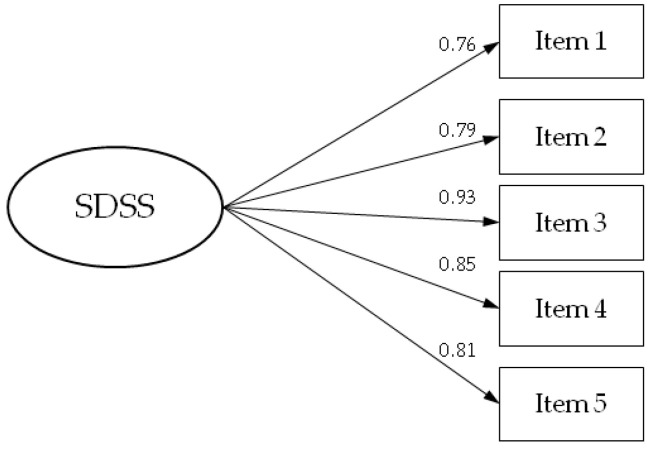
Standardized factor loadings for the five-item single-factor model of the SDSS questionnaire.

CFA was performed to evaluate the adequacy of the five-item single-factor model of the SDSS questionnaire (Figure 1, Table 4). The Chi-square goodness-of-fit test was not statistically significant (χ^2^ = 10.094, df = 5, *p* = 0.073), indicating no substantial difference between the model and the observed data. The RMSEA value was 0.123 (90% CI: 0.000–0.234). Incremental fit indices were high, with IFI of 0.979, CFI of 0.978, and TLI of 0.956, supporting an acceptable model fit. While the RMSEA value exceeded the commonly suggested threshold, all other fit indices indicated that the single-factor model provided an adequate representation of the data. Standardized factor loadings for the SDSS items are presented in Figure 1. All factor loadings were statistically significant, ranging from 0.76 to 0.93, indicating strong relationships between each item and the underlying construct (Figure 1, Table 4).

**Figure 2:**
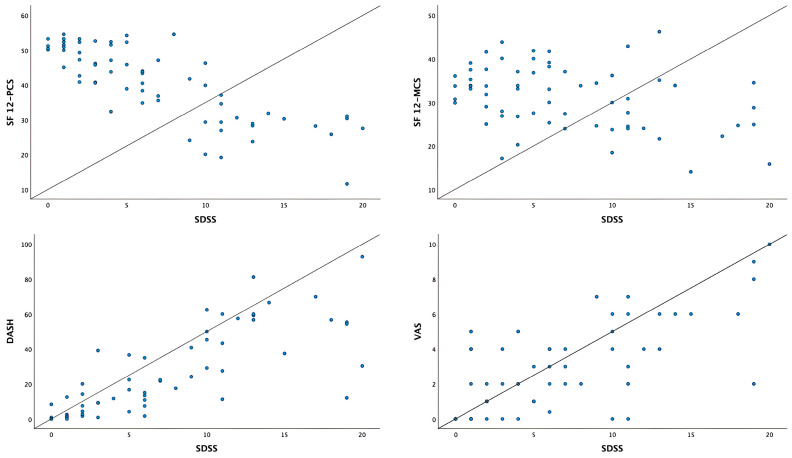
Correlations between SDSS and SF-12 PCS, SF-12 MCS, DASH and VAS.

Pearson’s correlation coefficients for the SDSS, DASH, SF-12 PCS, SF-12 MCS, and VAS are presented in Table 5. The SDSS score showed a strong positive correlation with the DASH score (r = 0.779) and VAS score (r = 0.702). A strong negative correlation of SDSS was found with SF-12 PCS (r = −0.802), while the correlation with SF-12 MCS was moderate and negative (r = −0.363). The DASH score was strongly and negatively correlated with SF-12 PCS (r = −0.768) and strongly positively correlated with VAS score (r = 0.819). SF-12 PCS was strongly and negatively correlated with VAS score (r = −0.730) (Table 5). These correlations are illustrated in Figure 2.

## 4. Discussion

Building on the need for culturally adapted patient-reported outcome measures, this study indicates that the Serbian SDSS demonstrates robust psychometric performance and reliably captures a unidimensional construct of functional disability in Dupuytren’s disease. In our analysis, the Serbian version of the SDSS showed excellent internal consistency (Cronbach’s α = 0.914), exceeding the values reported in the original validation by Mohan et al. (Cronbach’s α = 0.87) and in the Danish validation by Bendixen et al. (Cronbach’s α = 0.76) [18,26]. CFA further supported this interpretation, confirming a stable one-factor structure with strong item loadings ranging from 0.76 to 0.93. Beyond its internal structure, the SDSS also demonstrated strong external validity when compared with established outcome measures. In our study, the SDSS showed a markedly stronger correlation with the full DASH (r = 0.779) than previously reported between SDSS and QuickDASH in the original validation by Mohan et al. (r = 0.598) [16]. Our results reinforce this perspective, as we also observed strong associations between SDSS and symptomatic measures, including VAS pain (r = 0.702) and the physical component of the SF-12 (PCS; r = −0.802). Mental health in affected individuals remains largely preserved despite marked physical limitations, as demonstrated by the moderate negative correlation with the SF-12 mental component (MCS; r = −0.363) observed in our study, which aligns with previous research showing similarly weak associations between SDSS scores and mental health measures [21]. Analysis of individual SDSS items provided additional insight into patient experiences. While many participants reported only minor or modest inconvenience across domains, a significant proportion described severe difficulties in personal care (14.7%), hobbies (14.7%), and work or social interactions (11.8%). Wilburn et al. demonstrated through qualitative interviews that patients frequently report difficulties with dressing, gripping, and personal care, all of which directly compromise independence and quality of life [29]. Similarly, a large registry study confirmed that patients treated with fasciectomy, collagenase injection, or needle fasciotomy continued to experience symptoms such as stiffness, numbness, and cold sensitivity, which directly affect everyday and recreational activities [30]. These observations align with our findings that a notable proportion of patients reported severe difficulties in hobbies, occupational tasks, and social interactions. Furthermore, the systematic review by Bradet-Levesque et al. emphasized that generic upper-extremity questionnaires such as DASH may underestimate the disease-specific limitations associated with Dupuytren’s disease, whereas condition-specific instruments like the SDSS provide a more precise and comprehensive assessment of its impact on daily functioning [17].

The Danish validation reported a standardized response mean of 1.96, confirming that the SDSS is highly sensitive to treatment effects, while a more recent Danish study established a minimal clinically important difference (MCID) of 1.5–1.62 points, providing an important reference value for interpreting longitudinal outcomes [31]. The study of Fletcher et al. further reinforce the role of collagenase Clostridium histolyticum as an effective treatment option for Dupuytren’s contracture, 68% of patients reported being satisfied or very satisfied with their outcome at one year, and the majority of those with prior surgical treatment (85.7%) stated they would prefer collagenase injection over surgery. The overall magnitude of improvement exceeded the MCID previously defined for the SDSS, thereby confirming that the changes were clinically meaningful [32].

This study has certain limitations. It was carried out in a single-center setting with a relatively modest sample size. Future multicenter studies with larger samples are needed to confirm the psychometric properties of the Serbian SDSS.

Our validation of the Serbian SDSS provides an essential tool for clinicians and researchers in our region, ensuring that patient-reported outcomes can be reliably measured and compared internationally. These outcomes facilitate the integration of the SDSS into both clinical practice research and the clinical relevance of our item-level results and underscores the necessity of employing disease-specific instruments capable of capturing both the physical and psychosocial dimensions of disability in patients with Dupuytren’s disease. In clinical care, the SDSS can serve as a valuable tool for tailoring treatment strategies: patients with lower baseline scores may be more suitable for non-surgical interventions such as collagenase injections or radiotherapy, whereas those with higher scores may benefit more from surgical approaches. In research, the SDSS offers a powerful means of quantifying patient-centered outcomes, making it valuable for evaluating both established and novel therapies, and it could also be integrated into digital health platforms and remote monitoring systems to enable real-time outcome assessment and support more personalized care. By establishing the psychometric robustness of this Serbian version, our study closes an important gap and supports the wider adoption of standardized, patient-centered outcome measures in order to ensure more efficient treatment and quality of life in patients with Dupuytren’s contracture.

## 5. Conclusions

Overall, our results extend the existing body of evidence by demonstrating that the Serbian version of the SDSS maintains excellent reliability and validity, aligns with international findings, and captures unique dimensions of the disease that are not addressed by generic measures. By encompassing functional disability, quality of life, and psychosocial burden, the SDSS provides a comprehensive evaluation of patient outcomes in Dupuytren’s disease. Its adoption in both clinical and research contexts has the potential to enhance individualized patient care, support shared decision-making, and improve the comparability of treatment outcomes across studies and populations.

## Figures and Tables

**Table 1:** Characteristics of the study population.

Variables	*n* = 68
Age, mean ± sd	64.6 ± 9.5
Gender-male, n (%)	
Male	55 (80.9)
Female	13 (19.1)
SDSS total score, mean (95% CI)	7.1 (5.7–8.5)
DASH, mean (95% CI)	26.5 (20.2–32.9)
SF-12, mean (95% CI)	
PCS	47.0 (44.4–49.7)
MCS	42.1 (40.3–43.9)
VAS, median (25th–75th percentile)	2 (0.9–4.3)

**Table 2:** Participants’ responses to the SDSS items.

	No Problem	Minor Inconvenience	Modest Inconvenience	Definitely Troublesome	Severe Problem
How much trouble do you have with:					
Discomfort	17 (25.0)	25 (36.8)	11 (16.2)	10 (14.7)	5 (7.4)
Personal activities, e.g., washing face, dressing, washing hands, washing hair, putting on gloves	18 (26.5)	15 (22.1)	16 (23.5)	9 (13.2)	10 (14.7)
Domestic activities, e.g., holding a glass/cup, opening jars, eating, cooking.	23 (33.8)	18 (26.5)	15 (22.1)	6 (8.8)	6 (8.8)
Work/Social interaction, e.g., using the computer, writing, shaking hands, cosmetic appearance.	26 (38.2)	18 (26.5)	14 (20.6)	2 (2.9)	8 (11.8)
Hobbies, e.g., driving/cycling, racket sports, DIY, playing musical instruments, gardening.	20 (29.4)	26 (38.2)	4 (5.9)	8 (11.8)	10 (14.7)
Personal activities, e.g.,: washing face, dressing, washing hands, washing hair, putting on gloves	18 (26.5)	15 (22.1)	16 (23.5)	9 (13.2)	10 (14.7)

Data are presented as *n* (%).

**Table 3:** Internal consistency of SDSS questionnaire.

	Domains	Nº Items	Cronbach’s Alpha	Internal Consistency
SDSS	Single factor	5	0.914	Excellent

**Table 4:** Fit statistics for SDSS questionnaire.

	Chi-Squared Goodness of Fit	df	*p*	RMSEA (90% CI)	IFI	CFI	TLI
SDSS	10.094	5	0.073	0.123 (0.000–0.234)	0.979	0.978	0.956

df-degrees of freedom, *p*-value, RMSEA-root mean square error of approximation, IFI-incremental fit index, CFI-comparative fit index, TLI-Tucker–Lewis index.

**Table 5:** Correlation coefficients between SDSS, DASH, SF-12-PCS, SF-12-MCS and VAS score.

		DASH	SF-12		VAS
			PCS	MCS	
SDSS		0.779 *	−0.802 *	−0.363 *	0.702 *
DASH			−0.768 *	−0.349 *	0.819 *
SF-12	PCS			0.223	−0.730 *
	MCS				−0.231

* *p* ≤ 0.050.

## Data Availability

The datasets used and/or analyzed during the current study are available from the corresponding author on reasonable request.
